# P02.184. Abstract withdrawn

**DOI:** 10.1186/1472-6882-12-S1-P240

**Published:** 2012-06-12

## Abstract withdrawn

